# Retraction Note: Human blood type influences the host-seeking behavior and fecundity of the Asian malaria vector *Anopheles stephensi*

**DOI:** 10.1038/s41598-022-14546-7

**Published:** 2022-06-17

**Authors:** Shahmshad Ahmed Khan, Nur Faeza Abu Kassim, Cameron Ewart Webb, Muhammad Anjum Aqueel, Saboor Ahmad, Sadia Malik, Taimoor Hussain

**Affiliations:** 1 Department of Entomology, University College of Agriculture, University of Sargodha, Sargodha, Pakistan; 2 Department of Entomology, Pir Mehr Ali Shah Arid Agriculture University Rawalpindi, Rawalpindi, Pakistan; 3 129 Medical Entomology Laboratory, School of Biological Sciences, Universiti Sains Malaysia, 11800 Minden, Penang Malaysia; 4 Marie Bashir Institute for Infectious Diseases and Biosecurity, University of Sydney, Sydney, New South Wales Australia; 5 Department of Entomology, Faculty of Agriculture and Environment, The Islamia University of Bahawalpur, Bahawalpur, Pakistan; 6 Key Laboratory of Pollinating Insect Biology, Ministry of Agriculture, Institute of Apicultural Research, Chinese Academy of Agricultural Sciences, Beijing, 100081 China; 7 Fatima Jinnah Women University, Rawalpindi, Pakistan; 8 Department of Agronomy, Pir Mehr Ali Shah Arid Agriculture University Rawalpindi, Rawalpindi, Pakistan

Retraction of: *Scientific Reports* 10.1038/s41598-021-03765-z, published online 21 December 2021

The Authors have retracted this Article. After publication, concerns were raised about overlaps with a previously-published article by the research group of two contributing authors, Khan and Ahmad^1^. Specifically, Figs. 2, 3, 5, 7 and 8b overlap with Figs. 4, 5, 2, 6 and 7 in^1^ respectively. A review of images and data presented in these figures identified errors and discrepancies that could not be resolved and, therefore, authors have lost confidence in the integrity of the data.

All Authors agree to the retraction and its wording.

## References

[1] Khan SA, Ombugadu A, Ahmad S (2022). Host-seeking behavior and fecundity of the female Aedes aegypti to human blood types. Pest Manag. Sci..

